# Supplementary material for: Association between oral microbiome and seven types of cancers in East Asian population: a two-sample Mendelian randomization analysis
Source: Front Mol Biosci. 2023 Nov 21;10:1327893. doi: 10.3389/fmolb.2023.1327893 (PMC10702768; doi:10.3389/fmolb.2023.1327893)
Supplement: Supplementary file 1 [file DataSheet1.ZIP › Supplementary Materials/MR plots of saliva/saliva═╝/ovarian cancer/pheno.150_to_ovarian cancer_forest.pdf]

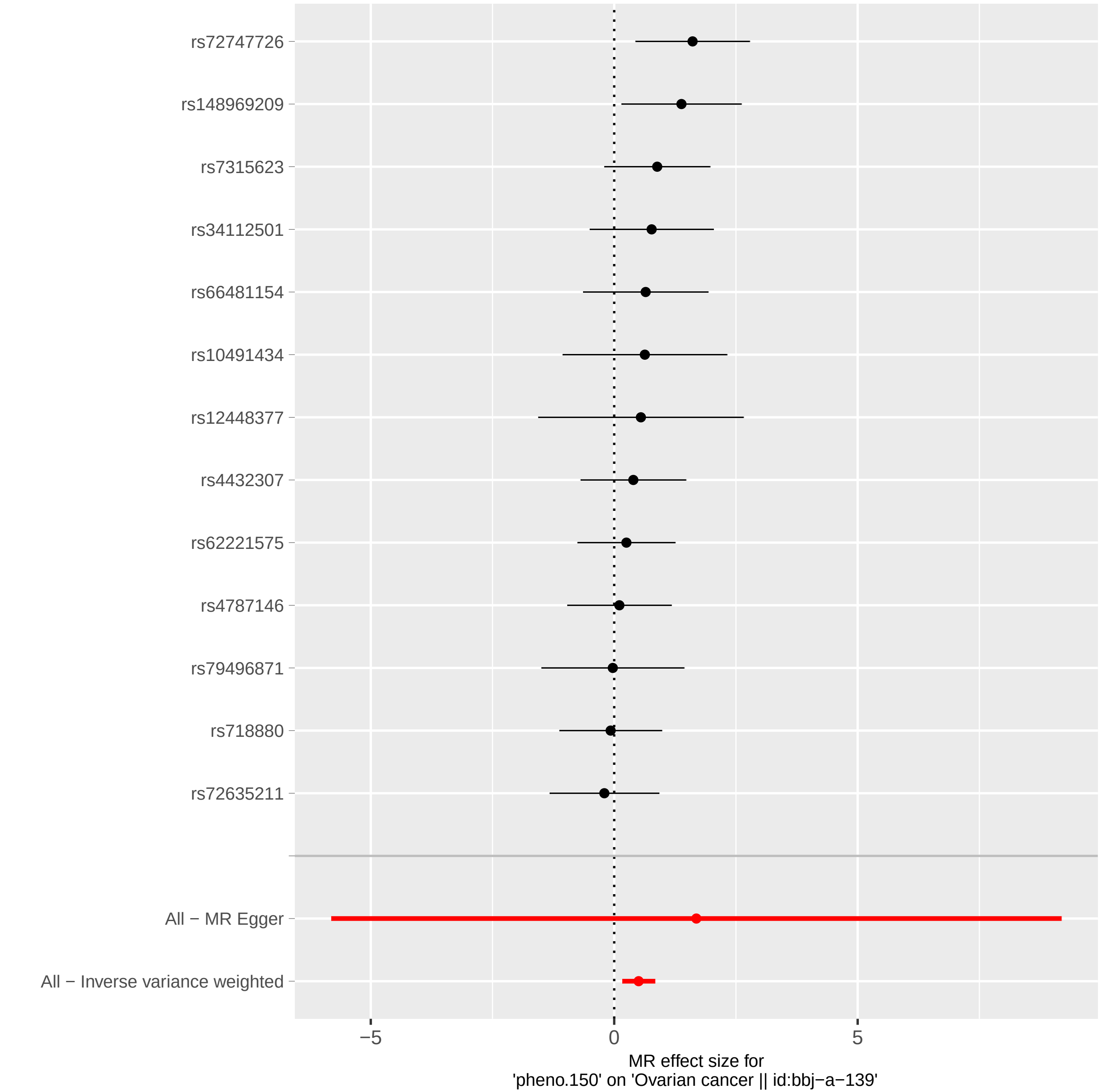

rs72747726
rs148969209
rs7315623
rs34112501
rs66481154
rs10491434
rs12448377
rs4432307
rs62221575
rs4787146
rs79496871
rs718880
rs72635211
All − MR Egger
All − Inverse variance weighted
−5
0
5
MR effect size for
'pheno.150' on 'Ovarian cancer || id:bbj−a−139'
